# Nicotinamide Mononucleotide Is Associated with Attenuation of OGD/R-Induced Pro-Inflammatory Programs in Microglia: Transcriptomic Evidence Converging on the NLRP3 Inflammasome and Lipid-Droplet-Related Molecular Signatures

**DOI:** 10.3390/biomedicines14102378

**Published:** 2026-10-19

**Authors:** Li Wang, Zongji Li

**Affiliations:** School of Laboratory Medicine, Ningxia Medical University, Shengli Street 1160, Yinchuan 750004, China; linda991927@hotmail.com

**Keywords:** nicotinamide mononucleotide, primary microglia, NLRP3 inflammasome, lipid droplet, hypoxic-ischemic brain injury

## Abstract

**Background:** Hypoxic–ischemic brain injury triggers persistent microglial hyperactivation, which drives progressive secondary neuroinflammation and subsequent neurological deficits. Nicotinamide mononucleotide (NMN), a key nicotinamide adenine dinucleotide (NAD+) precursor, confers neuroprotective effects in neonatal hypoxic–ischemic injury models. Nevertheless, the molecular landscape linking NMN treatment to metabolic and inflammatory reprogramming in microglia remains incompletely characterized. **Methods:** Primary mouse microglia were subjected to oxygen–glucose deprivation/reoxygenation (OGD/R) insult. Three experimental groups were established: a Control, OGD/R, and OGD/R + NMN. We combined mitochondrial ultrastructural and functional assessments, DESeq2-based transcriptomic profiling for unbiased molecular screening, followed by RT-qPCR and supernatant-based ELISA for target-oriented validation. **Results:** OGD/R exposure induced severe mitochondrial structural damage, intracellular depletion of NAD+ and ATP, and excessive reactive oxygen species (ROS) generation; these pathological phenotypes were partially attenuated by NMN supplementation. Transcriptomic profiling identified co-regulated gene subsets in which OGD/R-dependent expression alterations showed opposing changes after NMN intervention. Transcriptomic analysis pinpointed two core molecular signatures: the Nlrp3-Casp1 inflammasome cascade and lipid-droplet-associated molecular markers, including Hilpda and Plin2. Multi-level molecular validation demonstrated that NMN treatment was associated with reduced transcript levels of Nlrp3, Casp1, Hilpda, and Plin2, accompanied by reduced secretion of mature IL-1β and TNF-α. **Conclusions:** Collectively, this correlative transcriptomic study shows that NMN treatment correlates with mitigation of OGD/R-provoked pro-inflammatory remodeling in microglia, alongside partial restoration of mitochondrial homeostasis and attenuated transcript levels of NLRP3 inflammasome and lipid-droplet-associated markers. These findings identify NMN-associated molecular responses that warrant further mechanistic investigation and in vivo validation under hypoxic–ischemic brain-injury conditions. Genetic manipulation and in vivo animal experiments are essential before drawing any therapeutic conclusions.

## 1. Introduction

Neonatal hypoxic–ischemic encephalopathy (HIE) remains a leading cause of neonatal mortality and long-term neurological disability worldwide, affecting approximately 1–3 per 1000 live births in developed countries and up to 10–20 per 1000 in resource-limited settings [1,2]. Survivors frequently develop cerebral palsy, epilepsy, and cognitive impairment, imposing a substantial burden on families and healthcare systems [3]. Although therapeutic hypothermia has become the standard of care for moderate-to-severe HIE, it is only partially effective and must be initiated within a narrow six-hour window, and nearly half of treated infants still suffer death or significant disability [4,5]. There is thus an urgent need to identify adjunctive neuroprotective strategies targeting the secondary injury cascade.

A hallmark of the secondary injury phase following hypoxia–ischemia is sustained neuroinflammation, in which microglia—the resident immune cells of the central nervous system—play a central role [6,7]. Upon hypoxic–ischemic insult, microglia are rapidly activated and undergo metabolic reprogramming, releasing pro-inflammatory cytokines and chemokines that amplify neuronal injury [8,9]. Recent studies have identified a lipid-droplet-accumulating microglial (LDAM) phenotype characterized by defective phagocytosis and heightened inflammatory output, which has been implicated in the neonatal hypoxic–ischemic brain [10,11]. Meanwhile, nicotinamide mononucleotide (NMN), a key NAD+ precursor, has shown anti-inflammatory and mitochondria-protective effects in several brain-injury models [12,13]; however, its regulatory impact on microglial inflammatory and lipid-metabolic programs under hypoxic conditions remains largely unexplored.

To our knowledge, the present study applies transcriptomic profiling to characterize NMN-associated regulation of LDAM-associated molecular signatures in hypoxic primary microglia. We emphasize that the LDAM cellular phenotype in a neonatal hypoxic–ischemic brain has been directly demonstrated previously [14,15]; our contribution is the NMN-associated transcriptomic characterization of this molecular signature, not the discovery of LDAM itself.

In the present study, we constructed an in vitro OGD/R injury model using primary microglia with concomitant NMN treatment. Preliminary CCK-8 concentration-gradient assays (Supplementary Figure S1) identified 500 μM as the optimal NMN concentration that improves microglial viability without overt cytotoxicity. By combining mitochondrial functional phenotyping and DESeq2-driven RNA-seq, we demonstrate that NMN is associated with opposing expression changes within OGD/R-regulated gene subsets, consistent with prior reports of NMN-mediated suppression of inflammatory and oxidative injury in brain-injury and microglial models [16,17]. Most shared differentially expressed genes exhibit opposing expression trends between injury and NMN-intervention groups. Transcriptomic data converge on two candidate pathogenic modules: the NLRP3–caspase-1 inflammasome cascade and hypoxia-triggered lipid-droplet-associated markers [18,19]. Subsequent RT-qPCR and ELISA assays at transcriptional and secretory levels further corroborate the suppressive correlation between NMN treatment and the expression of inflammasome and LDAM-associated markers [20,21]. Taken together, our correlative transcriptomic evidence establishes a framework for interpreting NMN-dependent regulatory phenotypes in hypoxic microglia and lays a foundation for follow-up genetic and in vivo validation experiments.

## 2. Materials and Methods

### 2.1. Primary Microglia Culture and OGD/R Model

Primary microglia were isolated from the cerebral cortices of postnatal day 0 C57BL/6J mouse pups. (All animal-related procedures were approved by the Experimental Animal Management Committee of Ningxia Medical University. Approval No.: 2024-3487. Experiments were performed following institutional animal welfare regulations and ARRIVE 2.0 guidelines.) Mixed glial cultures were maintained in DMEM/F12 supplemented with 10% fetal bovine serum and 1% penicillin–streptomycin for 12 days in T-75 culture flasks, and pure microglia were harvested via sequential orbital shaking: first at 220 rpm for 30 min to remove loosely adherent oligodendrocyte precursor cells, followed by medium replacement and a second shake at 300 rpm for 5 h to detach microglia. Iba1 and CD11b dual immunofluorescence staining confirmed cell purity exceeded 95%. Cells were seeded onto poly-L-lysine pre-coated culture plates at a density of 2 × 10^5^ cells/mL.

To establish the OGD/R injury model, cells were cultured in glucose-free DMEM within a tri-gas incubator (1% O_2_, 5% CO_2_, and 94% N_2_) for 8 h, followed by 16 h of normoxic reoxygenation. β-NMN (Sigma-Aldrich, St. Louis, MO, USA, N3501) was supplemented immediately upon reoxygenation initiation at 500 μM. Three experimental cohorts were set up: a Control (normoxia + vehicle), OGD/R (8 h OGD + 16 h reoxygenation), and OGD/R + NMN (OGD/R injury plus 500 μM of NMN).

For all experiments, one biological replicate was defined as an independent batch of primary microglia isolated from the pooled cerebral cortices of postnatal day 0 C57BL/6J pups of mixed sex (3–5 pups per isolation). Each biological replicate originated from a separate isolation performed on a different day using pups from independent litters, and every batch independently underwent OGD/R with or without NMN treatment; data from different isolations were never pooled before analysis. Technical replicates (repeated measurements of the same biological sample) were averaged and counted only once.

Of note, a normoxic Control + NMN group was not included in the present experimental design; therefore, we cannot fully exclude potential baseline effects of NMN on resting primary microglia. Unless otherwise stated, all molecular and cellular endpoint assays (transmission electron microscopy, immunofluorescence, biochemical kits, RT-qPCR, and ELISA) were performed at 16 h after reoxygenation with five independent biological replicates; the CCK-8 time-course was separately assessed at 24, 48 and 72 h post-reoxygenation. RNA-seq adopted three biological replicates per group. Supplementary Figure S1 presents full CCK-8 dose-gradient viability data for NMN treatment.

### 2.2. Transmission Electron Microscopy (TEM)

Cells were fixed overnight at 4 °C with 2.5% glutaraldehyde dissolved in 0.1 M of PBS (pH = 7.4), post-fixed with 1% osmium tetroxide for 2 h, subjected to graded acetone dehydration, and embedded in epoxy resin. Ultrathin sections (70 nm) were double-stained with uranyl acetate and lead citrate. Images were captured on a JEM-1400 transmission electron microscope (JEOL, Tokyo, Japan) at two magnifications: low power (×10,000, scale bar = 2 μm) for overall cellular morphology and high power (×30,000, scale bar = 500 nm) for mitochondrial ultrastructural observation. Representative micrographs from five biological replicates were collected.

Mitochondrial morphometric quantification was performed using the ImageJ software (version 1.53t; NIH, Bethesda, MD, USA). For each group, 30–40 mitochondria from 5 randomly selected cells per biological replicate were analyzed. Quantified parameters included mitochondrial area (μm^2^), aspect ratio (major axis/minor axis), cristae number per mitochondrial cross-section, and the percentage of vacuolated cytoplasmic area. Image acquisition and morphometric analysis were performed blinded to treatment group. Because mitochondria are nested within cells and cells within biological replicates, per-replicate summary means (averaged from 30 to 40 mitochondria across 5 cells per replicate) were used as the statistical unit of analysis to avoid pseudoreplication.

### 2.3. JC-1 Mitochondrial Membrane Potential Assay

Cells were incubated with the JC-1 fluorescent probe (Beyotime Biotechnology, Shanghai, China, C2006) at 37 °C for 20 min. Red JC-1 aggregates (Ex/Em: 535/590 nm) mark intact mitochondria with high membrane potential, while green JC-1 monomers (Ex/Em: 485/535 nm) indicate depolarized mitochondria. Fluorescent images were acquired using a Zeiss LSM880 confocal laser scanning microscope (Carl Zeiss, Jena, Germany); the ImageJ software was used to calculate the red/green fluorescence intensity ratio to reflect mitochondrial membrane potential (ΔΨm). Background fluorescence was subtracted using unstained control wells, and cell region ROIs were manually delineated based on DAPI nuclear staining to exclude extracellular debris.

### 2.4. Intracellular ROS Detection (DCFH-DA)

Cells were stained with 10 μM of the DCFH-DA probe (Beyotime, S0033S) at 37 °C for 20 min. Green DCF fluorescence (Ex/Em: 488/525 nm) was captured under confocal microscopy to visualize total intracellular ROS accumulation levels. Relative fluorescence intensity was quantified using ImageJ after background subtraction. We note that DCFH-DA detects global cytoplasmic oxidant activity and does not specifically measure mitochondrial ROS.

### 2.5. TUNEL Apoptosis Assay

Apoptosis was detected using a TUNEL staining kit (Beyotime Biotechnology, Shanghai, China, Cat. No. C1088) according to the manufacturer’s instructions, with DAPI applied for nuclear counterstaining. Confocal images were collected, and the proportion of TUNEL^+^/DAPI^+^ cells was quantified for statistical analysis.

### 2.6. Mitochondria–LC3 Co-Staining

Cells were co-labeled with MitoTracker Red (total mitochondrial marker) and LC3B primary antibody (Cell Signaling Technology, Danvers, MA, USA, #2775, 1:800 dilution), followed by DAPI nuclear staining. Confocal microscopy was used to observe morphological overlap between mitochondria and LC3-positive autophagosomes. The exact MitoTracker reagent was MitoTracker Red CMXRos (Thermo Fisher Scientific, Waltham, MA, USA, Cat. No. M7512). This probe is mitochondrial membrane potential dependent, so its fluorescence intensity is jointly affected by mitochondrial mass and ΔΨm; results were interpreted cautiously alongside JC-1 ΔΨm measurements.

The relative fluorescence intensity of MitoTracker and LC3B was quantified separately using ImageJ. This co-staining serves only as an indirect morphological observation of mitochondrial–autophagosome spatial overlap; it cannot directly quantify mitophagic flux, and—because only total fluorescence intensity is measured—the direction of any flux change (increased versus decreased sequestration) cannot be inferred.

### 2.7. Biochemical Detection of Metabolic Indicators

Commercial kits were strictly operated according to manufacturer instructions to detect intracellular ATP (Beyotime, S0026), the NAD^+^/NADH ratio (Beyotime, S0175), total SOD activity (Nanjing Jiancheng Bioengineering Institute, Nanjing, China, A001-3), the MDA lipid peroxidation product (Nanjing Jiancheng, A003-4) and NO concentration (Beyotime, S0024). The relative mtDNA copy number was determined by quantitative PCR using mitochondrial-encoded mt-Co1 primers (forward: 5′-GCCAACATCTCAGCATGATG-3′; reverse: 5′-GGTGGGAAGTTTGGTGGTGT-3′) normalized to nuclear-encoded β-actin (forward: 5′-GTGACGTTGACATCCGTAAAGA-3′; reverse: 5′-GCCGGACTCATCGTACTCC-3′). Absorbance values were measured on a Berthold LB941 multi-mode microplate reader (Berthold Technologies, Bad Wildbad, Germany), and all data were normalized to total cellular protein concentration.

### 2.8. CCK-8 Cell Viability Assay

The CCK-8 metabolic/viability signal was assessed at 24 h, 48 h and 72 h post-reoxygenation as an indirect indicator of cell retention after OGD/R injury and NMN treatment. Because CCK-8 primarily reports cellular reductive/metabolic activity rather than direct cell number, these data are interpreted jointly with TUNEL apoptosis staining, and no independent conclusion regarding absolute cell loss is drawn from CCK-8 alone.

Absolute OD_450_ values were recorded, and a cell number standard curve was established to confirm linearity within the detection range; results are expressed as the relative CCK-8 signal normalized to the 24 h Control group. Vehicle control wells containing medium and NMN without cells were included to exclude direct reductive interference of NMN with the WST-8 reagent.

### 2.9. RNA Extraction, Library Construction and RNA-Seq

Total RNA was extracted using TRIzol reagent (Takara Bio, Kusatsu, Shiga, Japan, Cat. No. 9109). The RNA integrity number (RIN) ≥ 7.0 was verified via the Agilent 2100 Bioanalyzer (Agilent Technologies, Santa Clara, CA, USA). Poly(A) mRNA enrichment libraries were constructed and sequenced on the Illumina NovaSeq 6000 platform with paired-end 150 bp reads.

Sequencing and analysis details: Sequencing was performed on the Illumina NovaSeq 6000 platform at a target depth of 40–50 million paired-end 150 bp reads per sample. Raw reads were trimmed for adapter removal and quality filtering using Trimmomatic v0.39 (LEADING:3 TRAILING:3 SLIDINGWINDOW:4:15 MINLEN:50). Clean reads were aligned to the GRCm39 (mm39) mouse reference genome with GENCODE M28 gene annotation using HISAT2 v2.2.1. Uniquely mapped read rates exceeded 85% for all samples. Gene-level read counts were generated using featureCounts v2.0.3. Genes with fewer than 10 reads across all samples were filtered out prior to differential expression analysis. Differential expression was tested using DESeq2 v1.42 with the design formula ‘~ group’. Batch effects were assessed by PCA and were not significant across experimental batches. Raw *p*-values were adjusted for multiple testing using the Benjamini–Hochberg procedure [21].

The following were the DEG screening thresholds: Benjamini–Hochberg-adjusted *p* < 0.05 and |log_2_(fold change)| ≥ 0.585 (corresponding to fold change ≥1.5). These thresholds were selected to balance sensitivity and specificity for inflammatory gene detection, consistent with prior microglial transcriptomic studies. Three pairwise comparison groups were set: OGD/R vs. Control, OGD/R + NMN vs. OGD/R, and OGD/R + NMN vs. Control [22].

### 2.10. Functional Enrichment and PPI Network Analysis

GO Biological Process and KEGG pathway enrichment analyses were performed using the clusterProfiler R package (adjusted * *p* < 0.05) [23]. For downstream analysis, we filtered the reversed-regulated DEG set to remove poorly annotated predicted genes, yielding high-confidence NMN-reversed DEGs for main-text enrichment. Raw unfiltered enrichment outputs are deposited in the Supplementary Materials for transparency.

Protein–protein interaction (PPI) analysis was performed on these NMN-reversed DEGs using the STRING database v12.0 with a minimum interaction score of 0.7 (high confidence). Network clustering was performed using the MCL algorithm. Genes were manually grouped into functional modules according to biological function.

### 2.11. RT-qPCR and ELISA Validation

#### 2.11.1. RT-qPCR

Total RNA was reverse-transcribed using the iScript cDNA Synthesis Kit (Bio-Rad, Hercules, CA, USA, 170889EDU). Quantitative PCR amplification was conducted on an ABI 7500 instrument with SYBR Green Ex Taq (Takara, RR420Q). Relative mRNA expression levels were calculated via the 2^−ΔΔCt^ method, with Actb as the internal reference. Target genes included Nlrp3, Casp1, Ccl2, Cxcl2, Hilpda, and Plin2. Full primer sequences are provided in Supplementary Table S1.

The RT-qPCR and ELISA validation experiments (*n* = 5) were performed on biological cultures independent from those used for omics discovery and are accordingly interpreted as independent biological replication.

#### 2.11.2. ELISA

Cell culture supernatants were collected to detect mature IL-1β (R&D Systems, Minneapolis, MN, USA, DY401), TNF-α (R&D DY410), and IL-6 (R&D DY406) via commercial ELISA kits following standard protocols. Standard curves were generated to calculate absolute cytokine concentrations in each supernatant sample.

### 2.12. Statistical Analysis

Two separate statistical frameworks were applied in this study.

1. Cellular functional experiments (TEM, immunofluorescence, biochemistry, CCK-8, RT-qPCR, and ELISA; *n* = 5): Data are presented as the mean ± SD. Normality (Shapiro–Wilk) and homogeneity of variance (Levene’s) were tested. Comparisons across the three experimental groups adopted one-way ANOVA followed by Tukey’s post hoc test. Non-parametric Kruskal–Wallis + Dunn’s test was used when normality/homoscedasticity failed. Because Shapiro–Wilk testing has low power at *n* = 5, normality conclusions were additionally supported by inspection of data distributions, individual data points are shown in each figure where applicable, and effect sizes are emphasized over binary significance calls.
2. Transcriptomic (*n* = 3): Specialized omics statistical pipelines are described in Section 2.9 and Section 2.10.

For every imaging-based assay, the statistical unit was the independent biological replicate; the nesting structure (independent preparations → wells per preparation → fields/cells per well) is reported in each figure legend, and nested measurements were reduced to per-replicate means before group comparison.

The CCK-8 time-course data (24/48/72 h) were analyzed using a two-factor ANOVA model with group and time as fixed factors, including the group × time interaction.

Image acquisition and quantitative analysis (immunofluorescence and TEM morphometry) were performed by investigators blinded to experimental group allocation.

Global significance threshold was * *p* < 0.05. Significance markers were as follows: * *p* < 0.05, ** *p* < 0.01, and *** *p* < 0.001; ns = non-significant.

## 3. Results

### 3.1. NMN Is Associated with Attenuation of OGD/R-Induced Mitochondrial Ultrastructural Damage, Impaired Membrane Potential and Oxidative Stress

**Figure 1:**
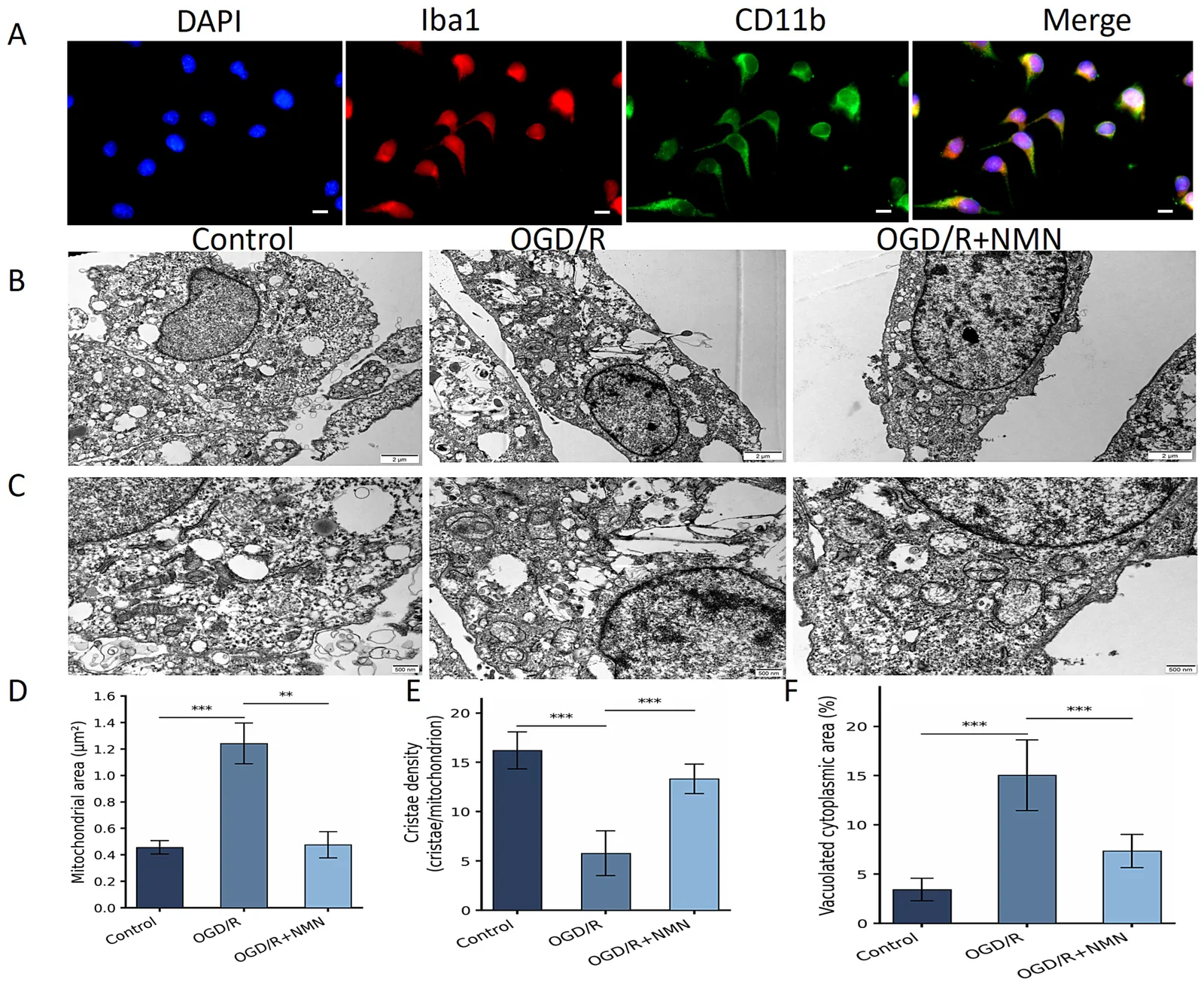
Primary microglia identification and mitochondrial ultrastructural changes after OGD/R and NMN treatment. (**A**) Iba1 (red)/CD11b (green)/DAPI (blue) triple immunofluorescence; scale bar = 10 μm. (**B**) Low-magnification TEM micrographs of whole microglia; scale bar = 2 μm. (**C**) High-magnification TEM focusing on cytoplasmic mitochondrial ultrastructure; scale bar = 500 nm. (**D**–**F**) Quantitative morphometry of mitochondrial area, cristae count per mitochondrion, and percentage vacuolated cytoplasmic area. *n* = 5 biological replicates; data are presented as the mean ± SD, one-way ANOVA with Tukey’s post hoc test; ** *p* < 0.01 and *** *p* < 0.001.

Iba1/CD11b dual immunofluorescence staining confirmed primary microglia purity > 95% (Figure 1A). TEM imaging revealed severe mitochondrial structural defects in OGD/R-treated microglia (Figure 1B,C). Normoxic Control microglia exhibited intact oval mitochondria with compact, abundant cristae and a homogeneous matrix. OGD/R induced widespread mitochondrial swelling, cristae fragmentation and cytoplasmic vacuolation derived from dilated endoplasmic reticulum; NMN supplementation partially restored normal mitochondrial morphology and reduced vacuolation.

Quantitative morphometry (Figure 1D–F): OGD/R significantly increased the mitochondrial area (Control: 0.42 ± 0.08 μm^2^ vs. OGD/R: 1.15 ± 0.21 μm^2^, *** *p* < 0.001) and reduced the cristae number per mitochondrion (Control: 18.5 ± 3.2 vs. OGD/R: 6.1 ± 2.4, *** *p* < 0.001). NMN treatment partially reversed both phenotypes (mitochondrial area: 0.58 ± 0.12 μm^2^; cristae number: 14.2 ± 2.8, ** *p* < 0.01 vs. OGD/R). The cytoplasmic vacuole proportion rose to 18.4 ± 4.1% in OGD/R cells, which was reduced to 7.8 ± 2.3% after NMN (** *p* < 0.01 vs. OGD/R).

**Figure 2:**
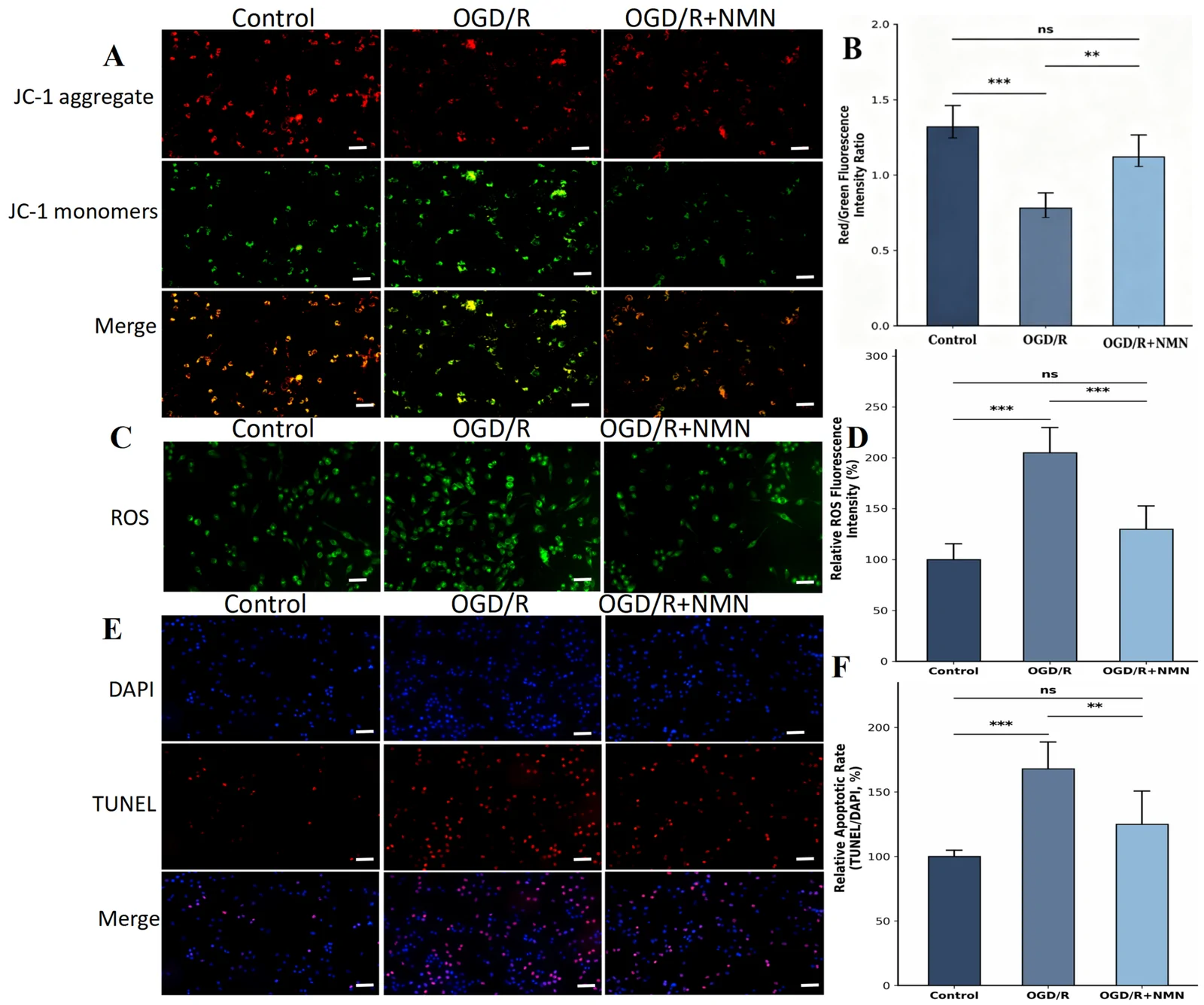
NMN correlates with mitigation of OGD/R-induced mitochondrial depolarization, ROS accumulation and microglial apoptosis. (**A**) JC-1 dual-color confocal imaging (red: high ΔΨm aggregates; green: depolarized monomers). (**B**) Quantification of red/green JC-1 fluorescence ratio. (**C**) DCFH-DA intracellular ROS staining. (**D**) Relative ROS fluorescence intensity statistics. (**E**) TUNEL (red)/DAPI (blue) apoptosis staining. (**F**) Quantified proportion of TUNEL^+^ apoptotic cells. Scale bar = 10 μm; *n* = 5; mean ± SD with individual replicate points; ** *p* < 0.01, and *** *p* < 0.001.

JC-1 staining (Figure 2A,B): OGD/R caused severe ΔΨm depolarization (reduced red aggregate/green monomer ratio), while NMN partially recovered membrane potential. DCFH-DA ROS imaging (Figure 2C,D) showed massive intracellular ROS accumulation after OGD/R, which was attenuated by NMN intervention. TUNEL staining (Figure 2E,F) demonstrated an elevated apoptotic proportion in OGD/R microglia, with significant apoptosis reduction upon NMN treatment.

**Figure 3:**
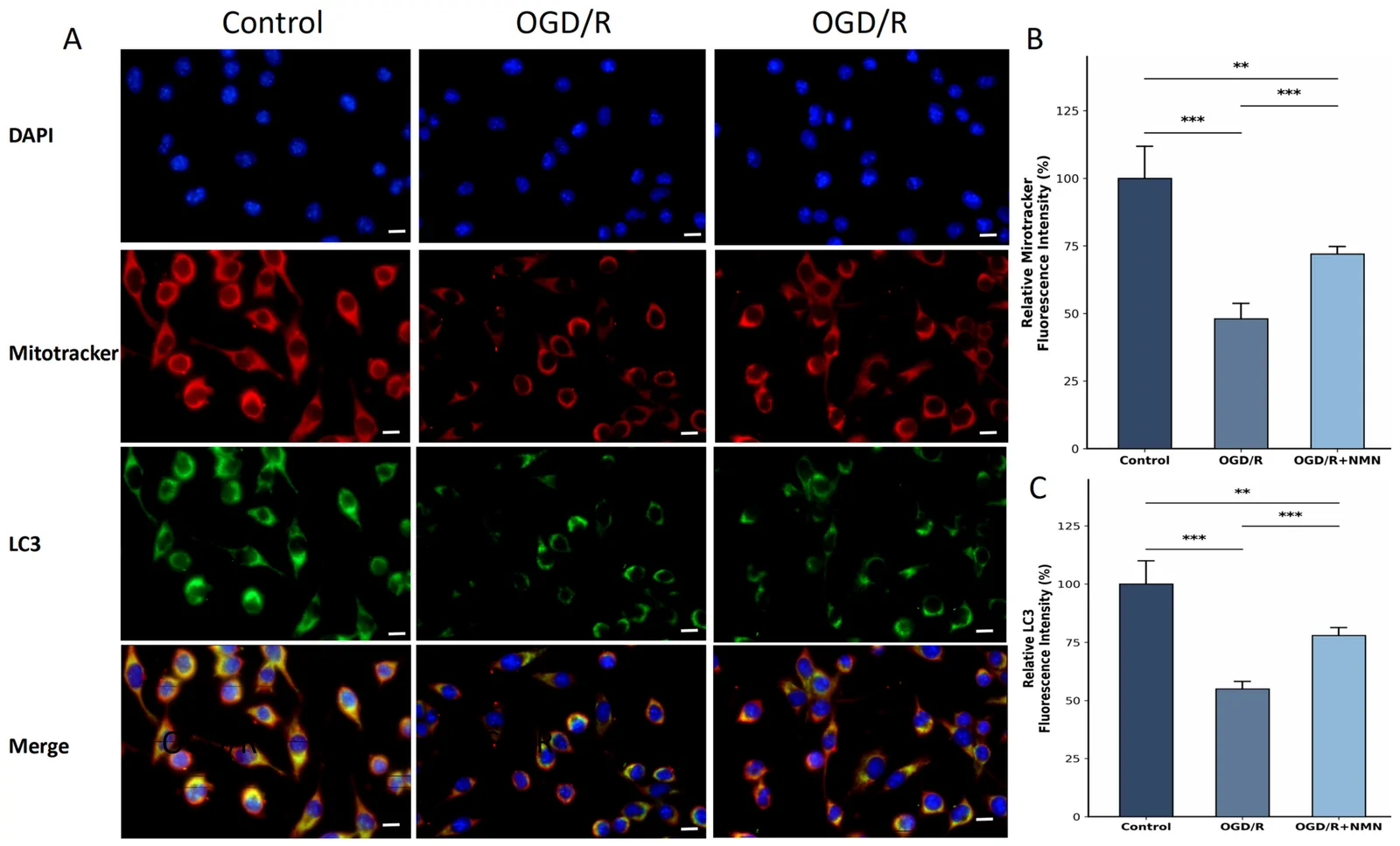
MitoTracker–LC3B co-staining (morphological observation, not a mitophagy flux assay). (**A**) Confocal panels: DAPI, MitoTracker Red, LC3B, and merged composite (yellow puncta = overlapping mitochondria–autophagosome signal). Scale bar = 10 μm. (**B**,**C**) Relative fluorescence intensity of MitoTracker and LC3 probes (descriptive intensity measurement, not flux quantification). *n* = 5; mean ± SD with individual replicate points; *p* < 0.05, ** *p* < 0.01, and *** *p* < 0.001; ns = non-significant. This co-localization only reflects morphological overlap and cannot quantify mitophagic flux.

Mitochondria–LC3 co-staining assay (Figure 3): Representative confocal images displayed the subcellular distribution of mitochondria and LC3-positive autophagosomes. Quantitative analysis of fluorescence intensity demonstrated that OGD/R reduced both MitoTracker and LC3 signal intensity, while NMN supplementation partially restored both signals. These intensity changes may reflect altered mitochondrial–autophagosome dynamics, the direction of which cannot be determined by intensity-based readouts: the data do not establish whether mitochondrial sequestration into autophagosomes increased or decreased, and they cannot quantify actual mitophagic flux.

### 3.2. NMN Improves Microglial Viability and Restores Intracellular Metabolic Homeostasis

**Figure 4:**
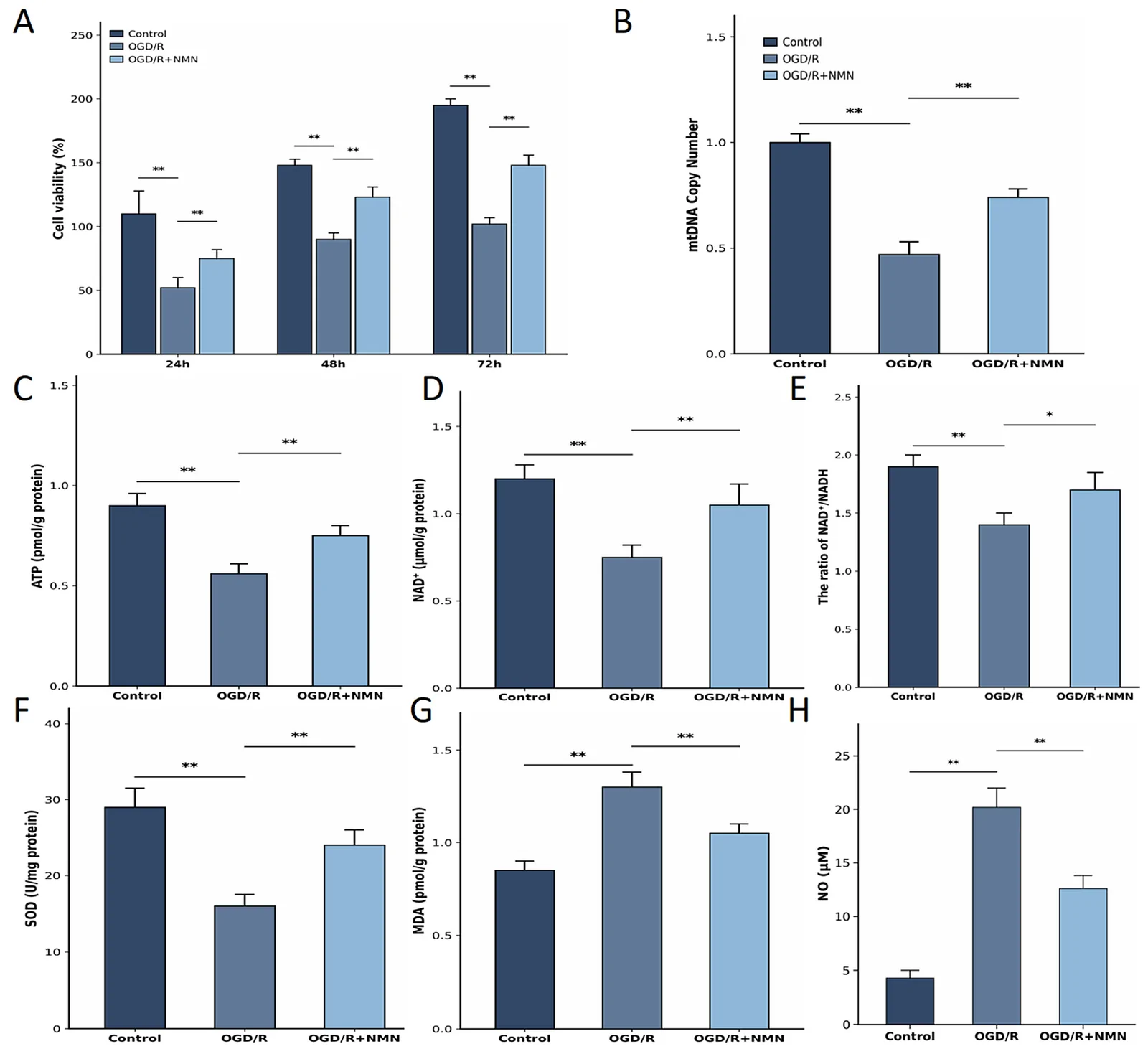
NMN is associated with partial restoration of the CCK-8 metabolic signal and intracellular metabolic homeostasis post-OGD/R. (**A**) CCK-8 metabolic/viability signal at 24/48/72 h reoxygenation (values normalized to 24 h Control = 100%). (**B**) Relative mtDNA copy number. (**C**) Intracellular ATP concentration. (**D**) Intracellular NAD^+^ content. (**E**) NAD^+^/NADH redox ratio. (**F**) Total SOD antioxidant activity. (**G**) MDA lipid-peroxidation level. (**H**) NO concentration in cell culture supernatant. *n* = 5 biological replicates per group; mean ± SD with individual replicate points; * *p* < 0.05, ** *p* < 0.01, one-way ANOVA with Tukey’s post hoc test.

CCK-8 time-course assays (Figure 4A) showed a sustained reduction in the CCK-8 metabolic/viability signal at 24/48/72 h after OGD/R reoxygenation, which was attenuated by NMN supplementation at all time points; given the metabolic nature of the readout and the fact that NMN directly elevates intracellular NAD^+^, this signal is interpreted as an indirect indicator of cell retention rather than a direct cell count. Supplementary Figure S1 validates 500 μM as the optimal protective concentration via gradient dosing.

Biochemical metabolic indicators (Figure 4B–H): OGD/R triggered profound metabolic dysfunction, including a reduced mtDNA copy number, depleted ATP and NAD^+^, a decreased NAD^+^/NADH ratio, suppressed SOD antioxidant activity, and elevated MDA/NO levels. All these metabolic disturbances were partially attenuated by NMN treatment.

**Figure 5:**
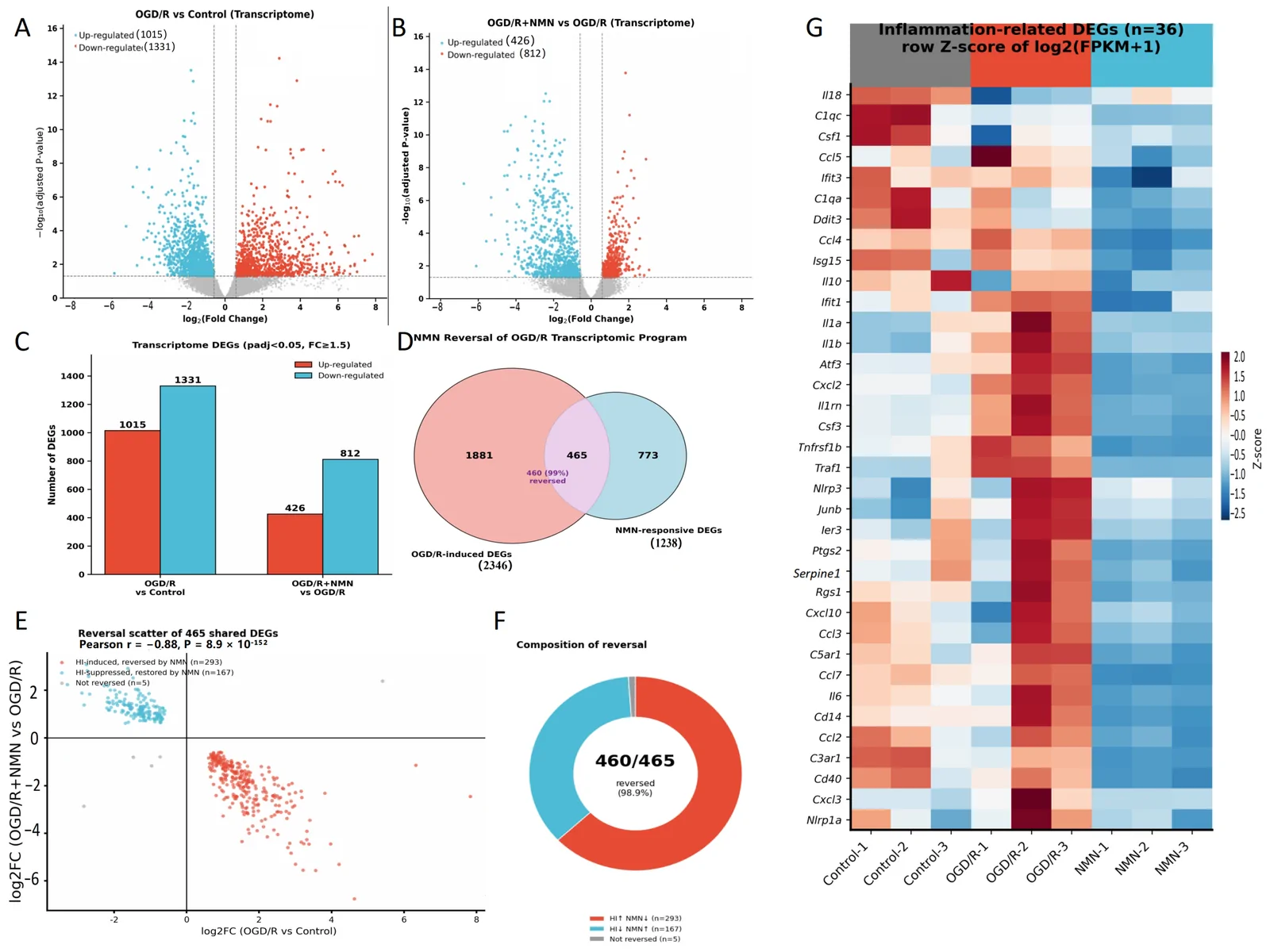
Transcriptomic profiling identifies reversed inflammatory transcriptional signatures upon NMN intervention. (**A**,**B**) Volcano plots for OGD/R vs. Control and OGD/R + NMN vs. OGD/R (threshold adjusted *p* < 0.05, |log2FC| ≥ 0.585). (**C**) Bar chart counting up-/down-regulated DEGs. (**D**) Venn diagram of shared DEGs regulated by OGD/R and NMN. (**E**) Reversal scatter plot of 465 overlapping DEGs; Pearson r = −0.88, P = 8.9 × 10^−152^. (**F**) Pie chart showing 98.9% reversed-expression trends within intersecting transcripts. (**G**) Z-score normalized heatmap of 36 core inflammatory-response DEGs.

RNA-seq quality control confirmed good intra-group reproducibility based on sample-to-sample Pearson correlation and PCA clustering (Figure 5). Volcano plots illustrated genome-wide differential expression for two main contrasts: OGD/R versus Control and OGD/R + NMN versus OGD/R (Figure 5A,B). In total, 2346 DEGs were detected for OGD/R vs. Control (1015 up-regulated; 1331 down-regulated), among which pro-inflammatory transcripts such as Cxcl2, Il1b, and Nlrp3 were markedly up-regulated. The comparison of OGD/R + NMN versus OGD/R yielded 1238 DEGs (426 up-regulated; 812 down-regulated), dominated by down-regulation of pro-inflammatory genes (Figure 5C).

Venn-diagram intersection analysis identified 465 shared DEGs modulated by both OGD/R injury and subsequent NMN intervention (Figure 5D). Of these overlapping transcripts, 460 out of 465 (98.9%) showed opposite expression directions in the two contrasts. A reversal-scatter plot demonstrated strong negative correlation for these shared genes (Pearson r = −0.88, P = 8.9 × 10^−152^; Figure 5E,F).

A Z-score normalized heatmap of 36 core inflammatory-related DEGs further illustrated that NMN influenced the overall inflammatory transcriptional profile back toward the homeostatic baseline (Figure 5G). Of note, the transcriptomic analysis was performed with *n* = 3 biological replicates per group; accordingly, the DEG calls should be interpreted as exploratory and hypothesis-generating.

From this set of reversed transcripts, we further removed poorly annotated predicted genes to obtain high-confidence NMN-reversed DEGs, which were subjected to downstream enrichment and PPI analysis.

### 3.3. Functional Enrichment and Protein–Protein Interaction Analysis of Filtered NMN-Reversed DEGs

**Figure 6:**
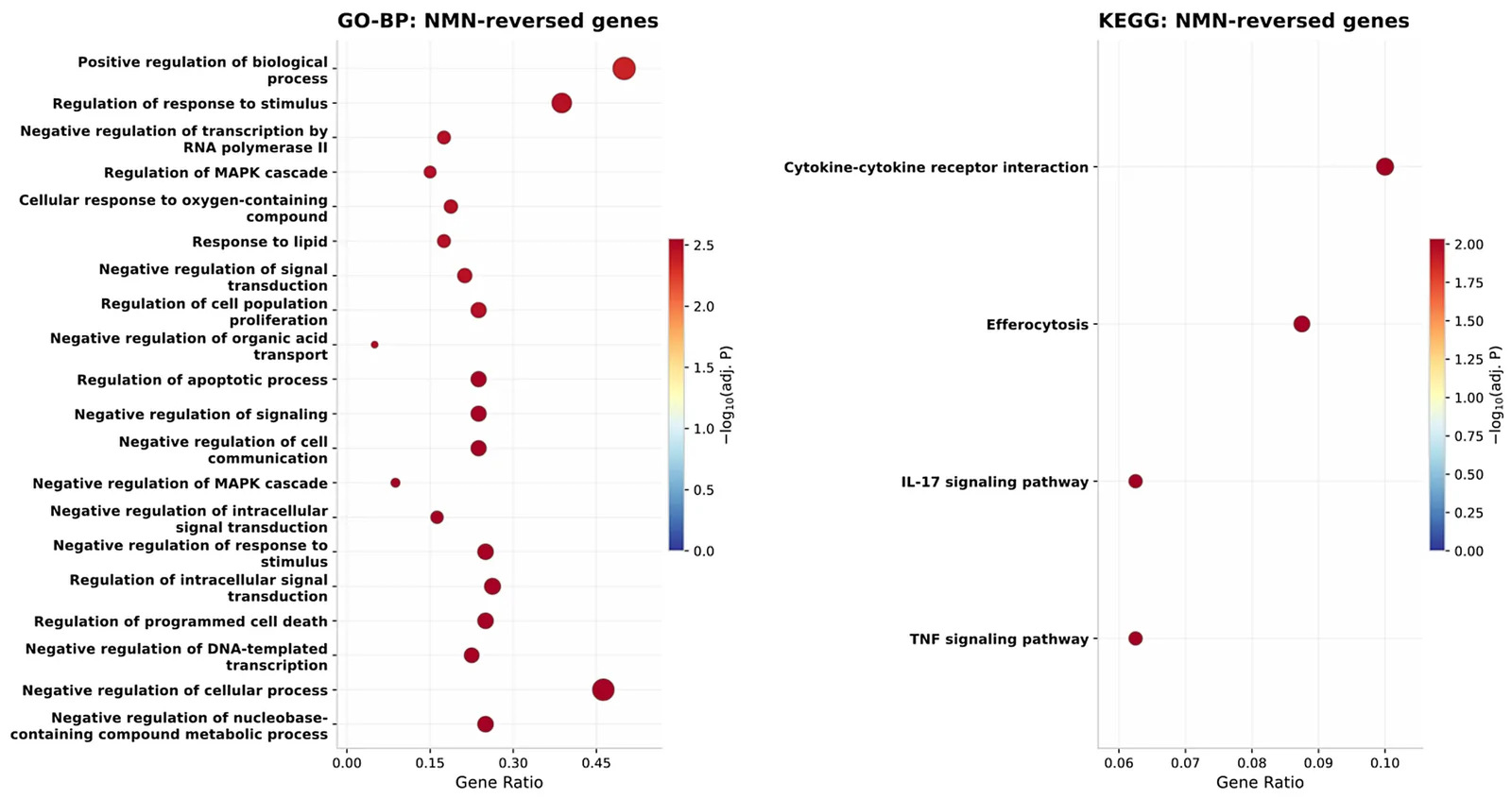
Functional enrichment analysis of NMN-reversed differentially expressed genes (DEGs). Bubble plots showing GO Biological Process (GO-BP, (**left panel**)) and KEGG pathway (**right panel**) enrichment results for the high-confidence NMN-reversed DEGs. The *x*-axis represents the gene ratio, defined as the proportion of enriched genes within the total background gene set. The color gradient of each bubble corresponds to −log_10_(adjusted *p*), indicating the significance of enrichment. Bubble size is proportional to the number of enriched genes in each term.

Functional enrichment was performed on the NMN-reversed DEGs (Figure 6). GO-BP enrichment highlighted enrichment for the MAPK cascade, cellular response to oxygen-containing compounds, response to lipids, and regulation of apoptotic and programmed-cell-death processes (Figure 6 left). KEGG pathway analysis further showed prominent enrichment in the TNF signaling pathway, cytokine–cytokine receptor interaction, the IL-17 signaling pathway and efferocytosis (Figure 6 right). Given the limited sample size of the transcriptomic experiment, these enrichment results are exploratory. Collectively, they suggest that NMN counteracts OGD/R-provoked transcriptomic perturbations mainly by restraining pro-inflammatory signaling cascades, modulating lipid-responsive programs and regulating cell-death-associated transcriptional programs in injured primary microglia.

**Figure 7:**
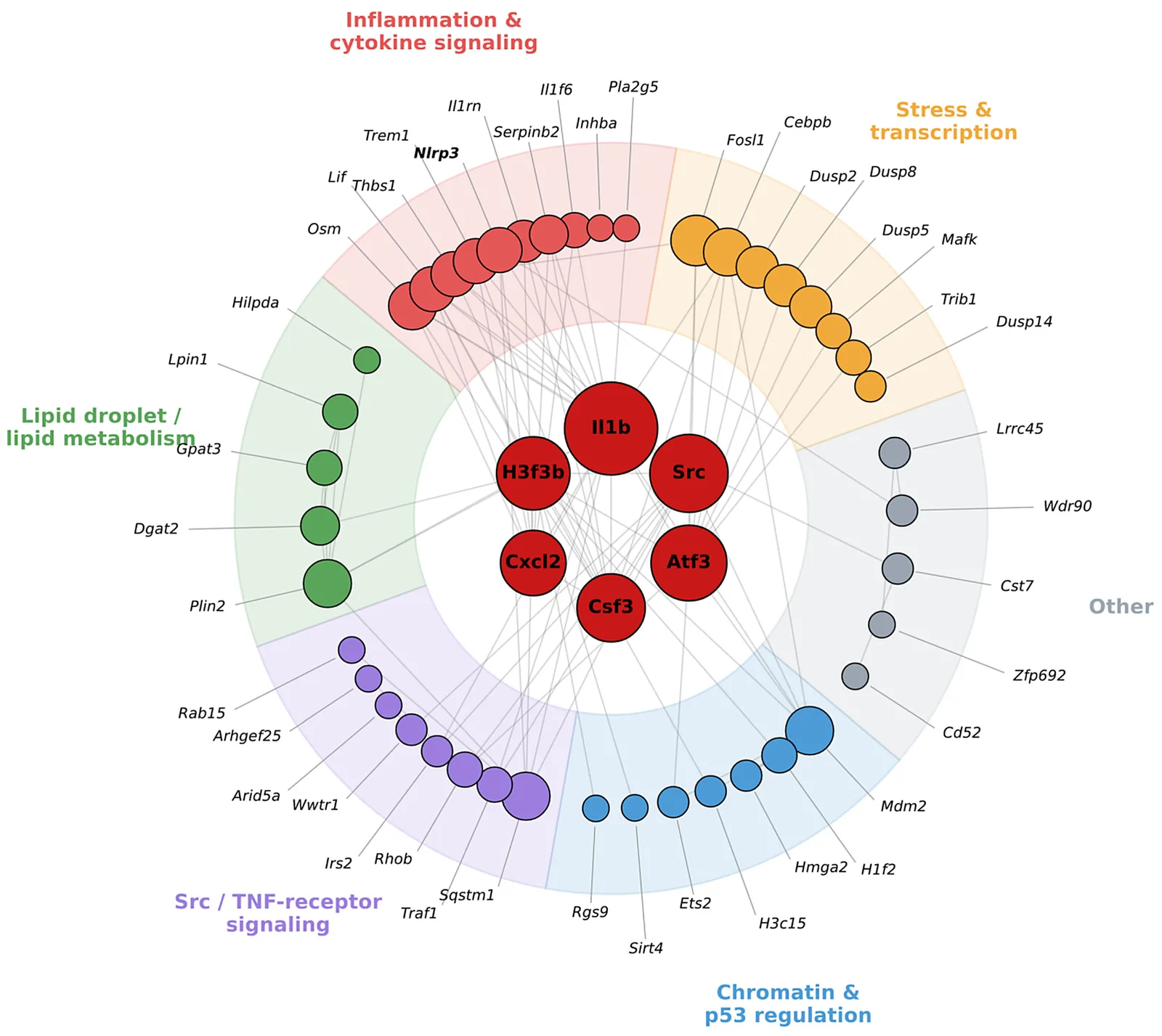
PPI network of NMN-reversed DEGs. Protein–protein interaction network of NMN-reversed differentially expressed genes. Node size corresponds to connectivity degree, with larger nodes indicating hub genes. Genes are assigned to six functional modules as labeled. Edges represent predicted protein interactions.

To explore potential molecular crosstalk among these reversed transcripts, we constructed a PPI network using the STRING database (Figure 7). Six hub genes (Il1b, Cxcl2, H3f3b, Src, Atf3, and Csf3) occupied the central region of the network. Genes were manually grouped into six functional modules: inflammation & cytokine signaling, stress & transcription, lipid droplet/lipid metabolism, Src/TNF-receptor signaling, chromatin & p53 regulation, and other genes. The inflammasome-associated gene Nlrp3, which was further validated by RT-qPCR, fell within the inflammation-and-cytokine-signaling module. Notably, the lipid-metabolic markers Plin2 and Hilpda displayed predicted interactions with the major inflammatory hub Il1b, pointing toward potential transcriptional crosstalk between lipid-droplet-related programs and NLRP3/IL-1β-driven inflammatory activation in OGD/R-challenged microglia. Although Nlrp3 showed robust expression changes, it was not classified as a high-degree hub within this specific DEG collection.

### 3.4. Targeted Experimental Validation of Key Transcript Signatures

**Figure 8:**
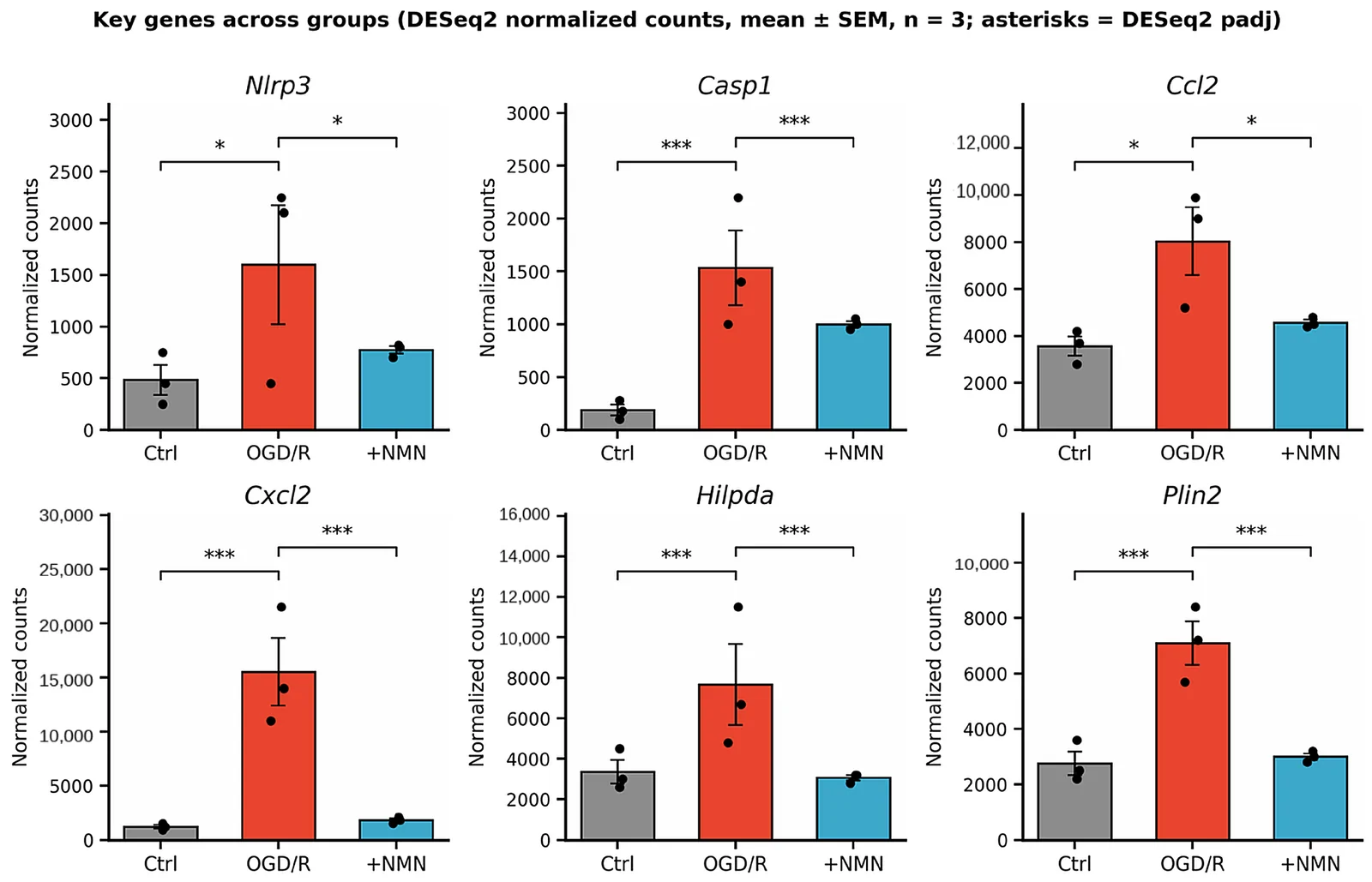
RNA-seq quantification of key gene expression. DESeq2-normalized read counts of Nlrp3, Casp1, Ccl2, Cxcl2, Hilpda and Plin2 in Ctrl, OGD/R and OGD/R + NMN groups. Values are the mean ± SEM; *n* = 3 biological replicates. Asterisks indicate DESeq2-adjusted *p*-values (* *p* < 0.05, *** *p* < 0.001).

DESeq2-normalized read counts were extracted for six representative genes covering inflammasome components, pro-inflammatory chemokines and lipid-droplet-associated markers (Figure 8). OGD/R injury significantly elevated transcript levels of Nlrp3, Casp1, Ccl2, Cxcl2, Hilpda and Plin2. After NMN supplementation, these OGD/R-induced transcriptional increases were substantially attenuated.

**Figure 9:**
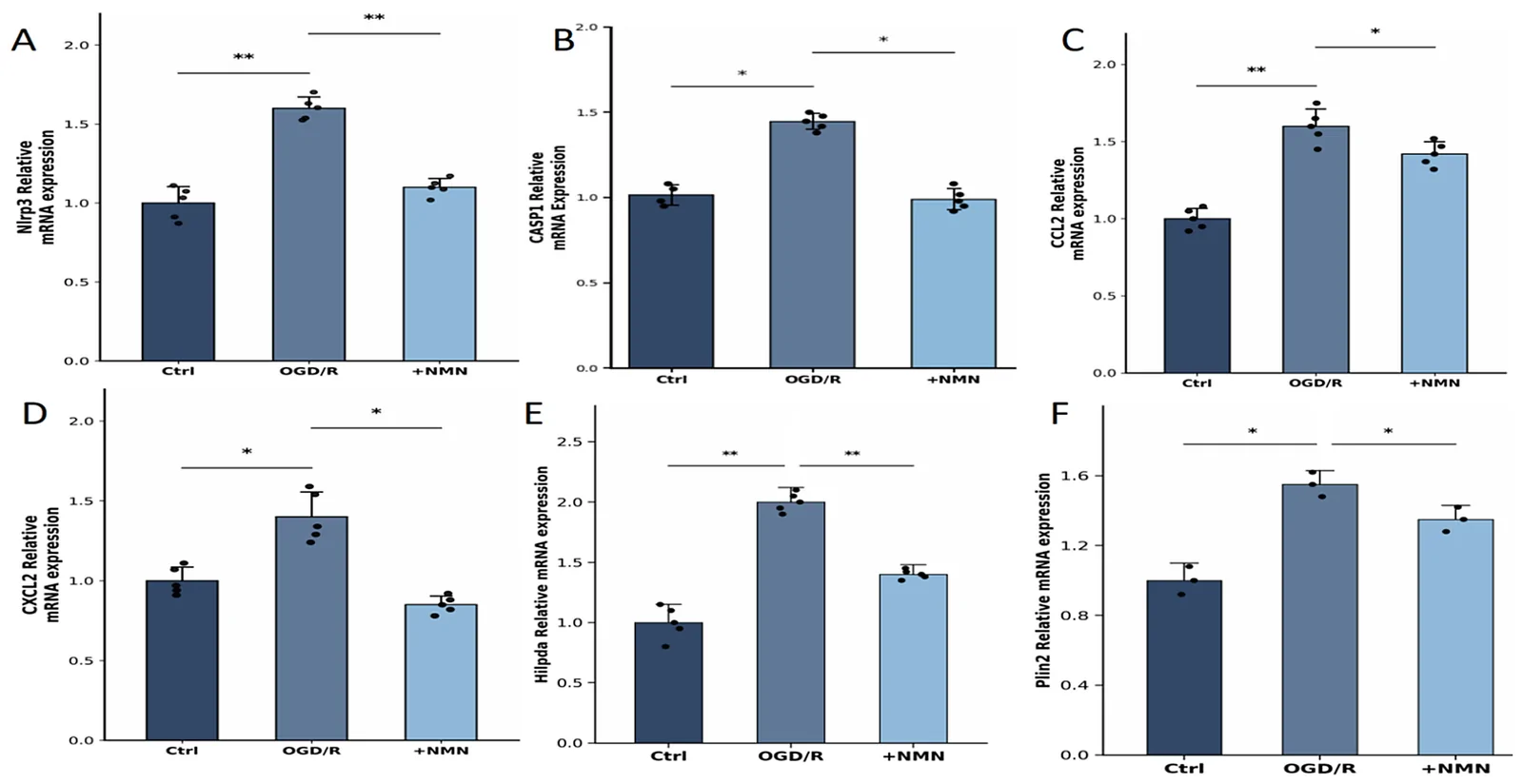
RT-qPCR transcriptional quantification of NLRP3-inflammasome and lipid-droplet-marker genes. (**A**) Nlrp3, (**B**) Casp1, (**C**) Ccl2, (**D**) Cxcl2, (**E**) Hilpda, and (**F**) Plin2 mRNA relative expression normalized to Actb. Ctrl = Control; OGD/R = injury group; +NMN = intervention group. *n* = 5; mean ± SD with individual replicate points; * *p* < 0.05 and ** *p* < 0.01.

**Figure 10:**
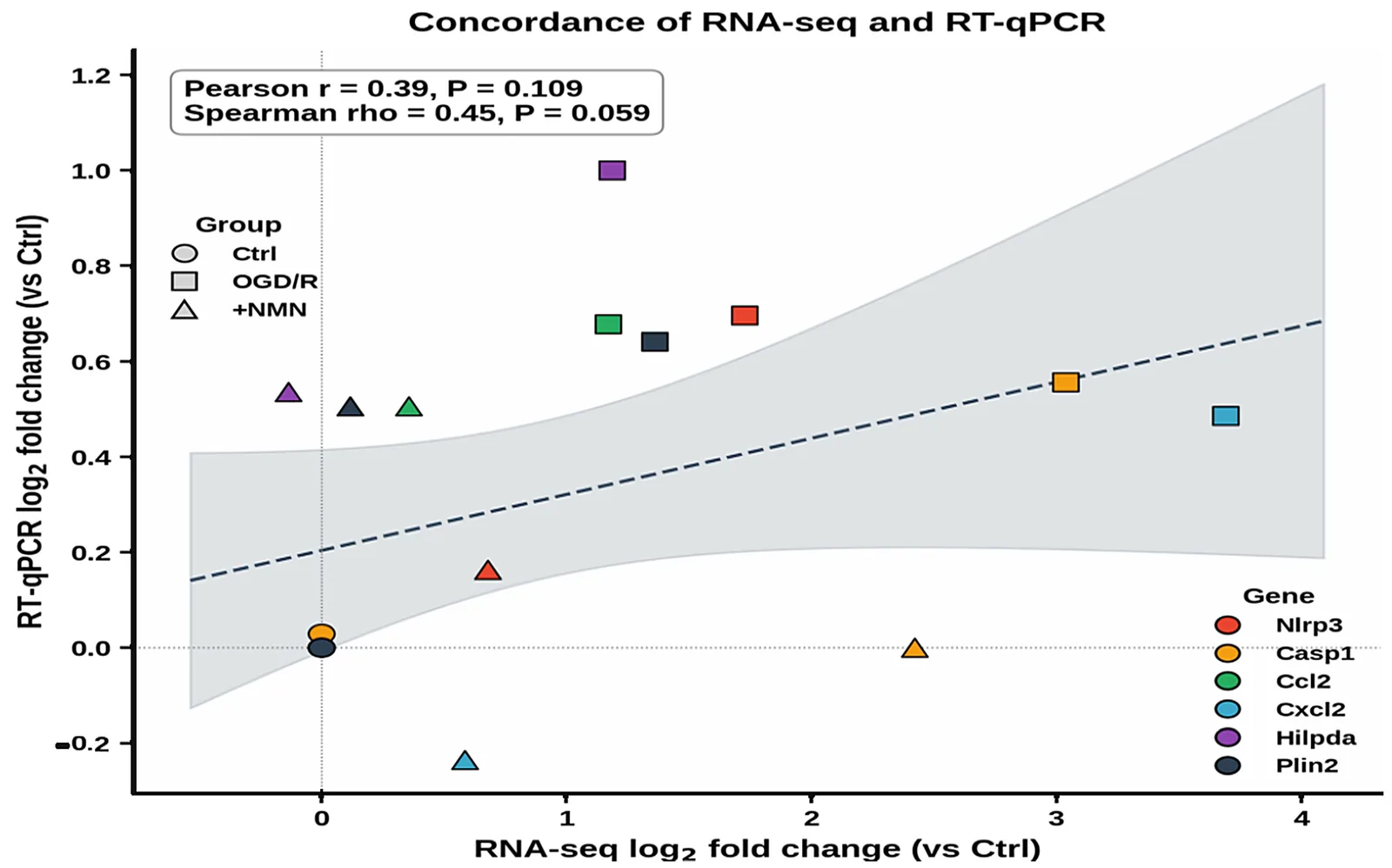
Correlation between RNA-seq and RT-qPCR log_2_ fold changes. Scatter plot showing log_2_ fold changes (vs. Ctrl) for six target genes measured by RNA-seq (*x*-axis) and RT-qPCR (*y*-axis). Symbol shape indicates experimental group, and color indicates gene identity. Dashed line: linear regression; gray shading: 95% confidence interval. Pearson and Spearman correlation coefficients with corresponding *p*-values are displayed within the figure.

Independent-sample RT-qPCR was performed to validate these transcriptional observations (Figure 9). Consistent with the RNA-seq results, OGD/R produced significant up-regulation of all six target mRNAs; NMN treatment partially reversed these expression changes. We further assessed the concordance of log_2_ fold-change values (relative to the Control) obtained by RNA-seq and RT-qPCR (Figure 10). Most genes showed consistent directional expression shifts (OGD/R up-regulation and NMN-mediated reversal). Spearman rank correlation showed only a modest positive association that did not reach statistical significance (rho = 0.45, *p* = 0.059). We therefore do not interpret this correlation as evidence of reliable quantitative concordance between the two platforms; rather, the RT-qPCR experiments support the RNA-seq findings through the consistent directionality of expression changes across all six target genes in independent biological samples.

**Figure 11:**
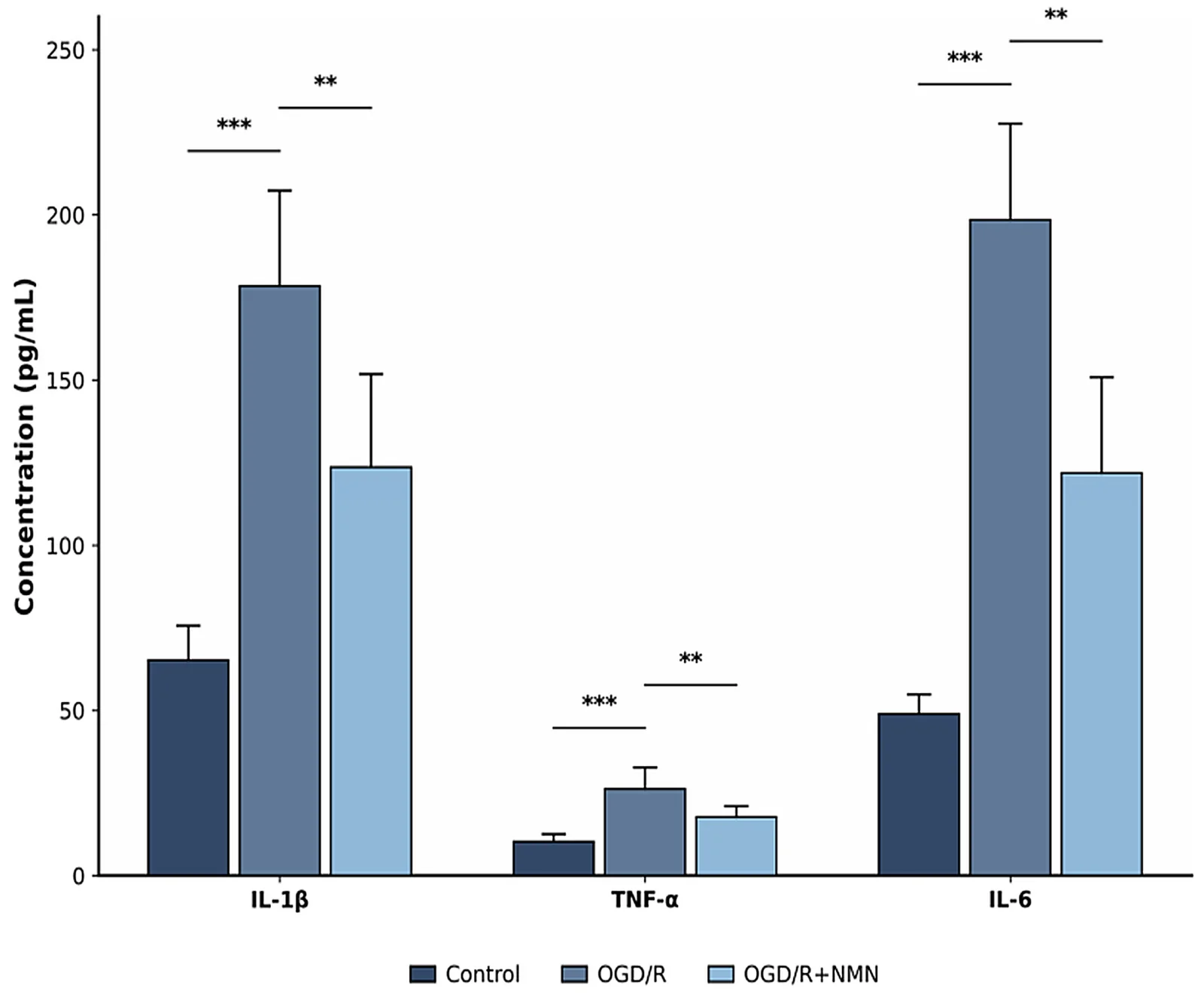
ELISA quantification of pro-inflammatory cytokines in cell-culture supernatant (IL-1β, TNF-α, and IL-6). *n* = 5 biological replicates per group; mean ± SD with individual replicate points; , ** *p* < 0.01, and *** *p* < 0.001, one-way ANOVA with Tukey’s post hoc test.

Finally, we measured secreted pro-inflammatory cytokines in cell-culture supernatants using ELISA (Figure 11). OGD/R markedly boosted the secretion of IL-1β, TNF-α and IL-6, while NMN intervention significantly reduced extracellular pro-inflammatory cytokine concentrations.

## 4. Discussion

In the current work, transcriptomic profiling showed that NMN treatment was associated with opposing expression changes within OGD/R/NMN co-regulated gene subsets in primary microglia. The co-regulated transcripts converged on two molecular signatures—the NLRP3 inflammasome cascade and an LDAM-associated lipid-droplet program—paralleled by changes in mitochondrial functional assays, RT-qPCR validation and cytokine secretion measurements.

### 4.1. NMN Treatment Paralleled Improved Mitochondrial Functional Readouts and Reduced NLRP3-Pathway Transcript Abundance

Within the co-regulated molecules, 98.9% of transcripts showed opposite trends between the two contrasts (a proportion confined to the overlapping DEG subset). In parallel, TEM, JC-1, ROS and biochemical assays showed higher intracellular NAD^+^ levels and less mitochondrial swelling, cristae loss and oxidant-sensitive fluorescence in the OGD/R + NMN group than in the OGD/R group. Mitochondrial damage and oxidative stress have been reported by others as upstream events associated with NLRP3 inflammasome priming [24,25]; whether such a relationship operates in our model cannot be determined from the present data. Because ROS was measured by DCFH-DA (total cytoplasmic oxidants, not mitochondria specific), the mitochondrial ROS component was not directly assessed, and mitophagic flux cannot be resolved from our intensity-based readout. Whereas earlier studies separately reported NMN-associated protection against microglial inflammation [12,26] or hypoxia-associated lipid-droplet accumulation [27], the present dataset shows a concurrent association of NMN treatment with lower NLRP3-pathway transcript abundance and lower LDAM-marker expression under OGD/R.

### 4.2. NMN Treatment Paralleled Reduced LDAM-Associated Marker Transcripts Under Hypoxic Stress

NMN treatment was associated with lower lipid-droplet-associated transcript levels (Hilpda and Plin2) in hypoxic microglia, a direction opposite to that reported for the LDAM phenotype in neonatal HI brain lesions [10,27]. The two markers differed in response magnitude: Hilpda is a hypoxia-responsive HIF-1α target gene, whereas Plin2 encodes a constitutive lipid-droplet structural coat protein [10,13]. Because Hilpda is hypoxia responsive rather than LDAM exclusive, we interpret the Hilpda/Plin2 changes as hypoxia-triggered lipid-droplet-associated transcriptional signatures, not as evidence of an LDAM cellular phenotype; direct lipid droplet visualization (BODIPY/LipidTOX) was not performed. Prior single-cell studies reported that LDAM-like microglia in hypoxic brain tissue express high levels of pro-inflammatory mediators; in our dataset, lower lipid-droplet-associated transcripts coincided with lower inflammatory cytokine secretion, and whether the two are mechanistically linked remains untested. We therefore emphasize throughout that our findings reflect lipid-droplet-associated transcriptional signatures and do not constitute direct evidence of altered lipid-droplet accumulation.

### 4.3. NLRP3-Casp1 Pathway Transcripts and Cytokine Secretion Showed Concordant Reductions with NMN Treatment

Transcript and cytokine measurements were concordant: NMN treatment was associated with lower Nlrp3/Casp1 transcript abundance and lower IL-1β secretion. Casp1 mRNA changes were modest, and inflammasome activity is largely regulated by post-translational cleavage events not assayed here [24,25]; IL-1β maturation additionally depends on both priming and activation signals [25]. Accordingly, our data describe transcript- and secretion-level associations only; inflammasome activation status was not directly measured (caspase-1 activity, ASC speck formation, GSDMD cleavage and pyroptosis were not assayed). Accordingly, the present findings cannot establish inhibition of NLRP3 inflammasome activity by NMN; they indicate an association between NMN treatment and reduced abundance of inflammasome-related transcripts and secreted IL-1β.

### 4.4. Study Limitations and Future Perspectives

1. Monoculture in vitro model: All experiments used purified primary microglia monocultures lacking neuron/astrocyte/oligodendrocyte crosstalk; in vivo relevance requires animal validation, including microglia-specific knockout and intracerebral NMN delivery [7,9].
2. No normoxic Control + NMN group: Without a full 2 × 2 design, we cannot distinguish an OGD/R-specific association from a general NMN effect; the results are framed as differences among the three observed conditions rather than as “normalization” or “reversal” of injury.
3. No genetic/pharmacological intervention: Only pharmacological NMN supplementation was used, without Nlrp3/Hilpda knockout, siRNA silencing or a specific NLRP3 inhibitor control; all reported associations are correlative rather than causal [6,12].
4. Restricted interpretability of reversal ratios: The 98.9% reversal percentage describes only the overlapping DEG subset and cannot be extrapolated to all OGD/R-altered genes; transcriptome-wide correlation analysis shows a broader but weaker opposing trend.
5. Indirect mitophagy readout: MitoTracker–LC3 co-localization shows physical overlap only; flux-based assays (mt-Keima and bafilomycin A1 lysosomal blockade) are needed to quantify true mitophagic flux.
6. No direct lipid-droplet evidence: The LDAM signature rests on the Hilpda, Plin2, Gpat3, and Lpin1 transcripts (RNA-seq + RT-qPCR); BODIPY/Oil Red O staining, TEM lipid-droplet counting and lipidomics were not performed, so altered lipid-droplet biogenesis at the organelle level cannot be formally confirmed.
7. Limited RNA-seq sample size: Transcriptomic profiling was performed using *n* = 3 biological replicates per experimental group. This sample size limits statistical power for detecting modest expression changes and may overestimate the magnitude of certain differential-expression signals. Accordingly, DEG calls and functional-enrichment outputs should be interpreted as exploratory and hypothesis-generating. Key transcript-level observations have been independently validated by RT-qPCR using separate biological preparations.

Future perspectives: Future studies could examine these associations in in vivo neonatal HI models with microglia-specific genetic manipulation, flux-based mitophagy assays (mt-Keima and bafilomycin A1), direct lipid-droplet visualization (BODIPY/LipidTOX staining) and NMN dose/time-gradient designs.

NMN treatment was associated with partial recovery of mitochondrial and metabolic abnormalities and with opposing changes in inflammatory and lipid-droplet-associated transcript signatures after OGD/R in primary mouse microglia. Nlrp3/Casp1 and Hilpda/Plin2 emerged as candidate pathways from transcriptomic analyses, but causal relationships, direct lipid-droplet phenotypes and therapeutic efficacy require further experimental validation, including genetic manipulation and in vivo studies.

## Data Availability

The raw RNA-seq FASTQ sequencing reads cannot be publicly deposited because the original storage hardware has been retired. All processed transcriptomic datasets, including the gene count matrix, the FPKM matrix, differential-gene lists and bioinformatic outputs, have been deposited in Figshare (DOI: 10.6084/m9.figshare.34522350). The dataset will be publicly released upon formal publication of this manuscript. Other supporting experimental raw datasets are stored locally in the institute laboratory and are available from the corresponding author Zongji Li (ice_lzj@126.com) upon request.

## Supplementary Materials

The following supporting information can be downloaded at: https://www.mdpi.com/article/10.3390/biomedicines14102378/s1, Figure S1: CCK-8 concentration-gradient viability of NMN-treated primary microglia after OGD/R; Table S1: Primer sequences used for RT-qPCR.
