# Supplementary material for: Nicotinamide Mononucleotide Is Associated with Attenuation of OGD/R-Induced Pro-Inflammatory Programs in Microglia: Transcriptomic Evidence Converging on the NLRP3 Inflammasome and Lipid-Droplet-Related Molecular Signatures
Source: Biomedicines. 2026 Oct 19;14(10):2378. doi: 10.3390/biomedicines14102378 (PMC13702338; doi:10.3390/biomedicines14102378)
Supplement: Supplementary file 1 [file biomedicines-14-02378-s001.zip › biomedicines-4621455-supplementary.pdf]

Supplementary Table S1. Primer sequences used for RT-qPCR analysis of microglial transcript levels.

All sequences are shown 5′→3′. Actb served as the internal reference gene, and relative expression was calculated using the 2^−ΔΔCt method.

Gene	Forward primer (5′→3′)	Reverse primer (5′→3′)
Actb	GTGACGTTGACATCCGTAAAGA	GCCGGACTCATCGTACTCC
Nlrp3	TCACAACTCGCCCAAGGAGGAA	AAGAGACCACGGCAGAAGCTAG
Casp1	GGCACATTTCCAGGACTGACTG	GCAAGACGTGTACGAGTGGTTG
Ccl2	TCTTGGTTCCCTGGCGTACTCT	GTGAGTGTCACTCTCCAGTTTGC
Cxcl2	CATCCAGAGCTTGAGTGTGACG	GGCTTCAGGGTCAAGGCAAACT
Hilpda	TGCTGGGCATCATGTTGACC	TGACCCCTCGTGATCCAGG
Plin2	GACAGGATGGAGGAAAGACTGC	GGTAGTCGTCACCACATCCTTC

Note: Actb primers are identical to the β-actin internal-control primers listed in Methods (section 2.7) and follow the Harvard PrimerBank design. Nlrp3, Casp1, Ccl2, Cxcl2, Hilpda and Plin2 primer pairs were selected from published/validated mouse SYBR Green primer sets (see Supplementary Methods). Primer pairs were verified by melting-curve analysis and agarose gel electrophoresis of single specific amplicons before use.

Supplementary Figure S1

A

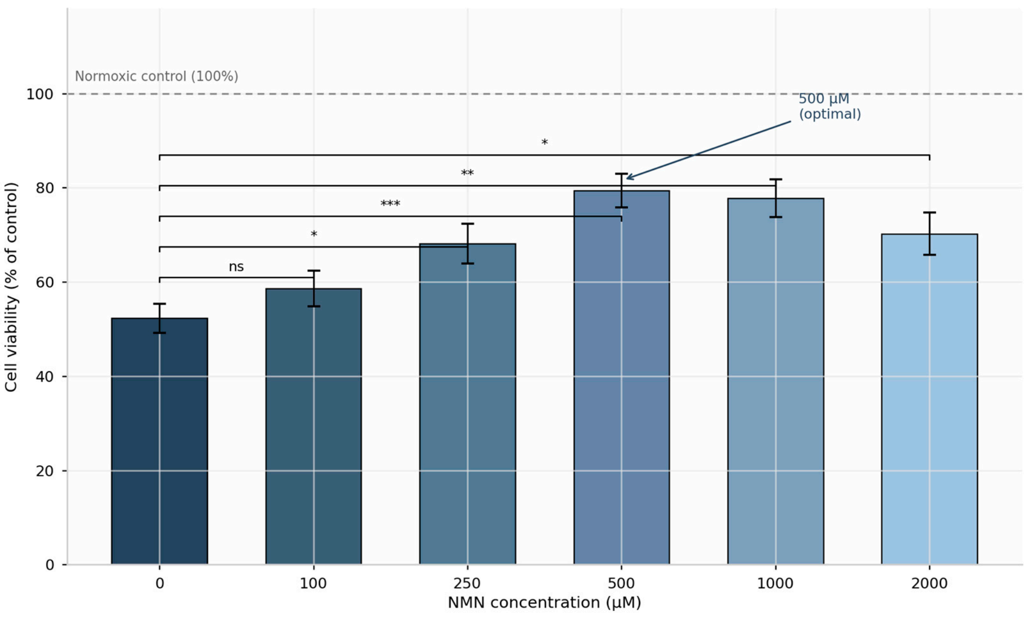

Figure S1: CCK-8 concentration-gradient viability of NMN-treated primary microglia after OGD/R
